# Differentiating ChatGPT-Generated and Human-Written Medical Texts: Quantitative Study

**DOI:** 10.2196/48904

**Published:** 2023-12-28

**Authors:** Wenxiong Liao, Zhengliang Liu, Haixing Dai, Shaochen Xu, Zihao Wu, Yiyang Zhang, Xiaoke Huang, Dajiang Zhu, Hongmin Cai, Quanzheng Li, Tianming Liu, Xiang Li

**Affiliations:** 1 School of Computer Science and Engineering South China University of Technology Guangzhou China; 2 School of Computing University of Georgia Athens, GA United States; 3 Department of Computer Science and Engineering University of Texas at Arlington Arlington, TX United States; 4 Department of Radiology Massachusetts General Hospital Boston, MA United States

**Keywords:** ChatGPT, medical ethics, linguistic analysis, text classification, artificial intelligence, medical texts, machine learning

## Abstract

**Background:**

Large language models, such as ChatGPT, are capable of generating grammatically perfect and human-like text content, and a large number of ChatGPT-generated texts have appeared on the internet. However, medical texts, such as clinical notes and diagnoses, require rigorous validation, and erroneous medical content generated by ChatGPT could potentially lead to disinformation that poses significant harm to health care and the general public.

**Objective:**

This study is among the first on responsible artificial intelligence–generated content in medicine. We focus on analyzing the differences between medical texts written by human experts and those generated by ChatGPT and designing machine learning workflows to effectively detect and differentiate medical texts generated by ChatGPT.

**Methods:**

We first constructed a suite of data sets containing medical texts written by human experts and generated by ChatGPT. We analyzed the linguistic features of these 2 types of content and uncovered differences in vocabulary, parts-of-speech, dependency, sentiment, perplexity, and other aspects. Finally, we designed and implemented machine learning methods to detect medical text generated by ChatGPT. The data and code used in this paper are published on GitHub.

**Results:**

Medical texts written by humans were more concrete, more diverse, and typically contained more useful information, while medical texts generated by ChatGPT paid more attention to fluency and logic and usually expressed general terminologies rather than effective information specific to the context of the problem. A bidirectional encoder representations from transformers–based model effectively detected medical texts generated by ChatGPT, and the *F*_1_ score exceeded 95%.

**Conclusions:**

Although text generated by ChatGPT is grammatically perfect and human-like, the linguistic characteristics of generated medical texts were different from those written by human experts. Medical text generated by ChatGPT could be effectively detected by the proposed machine learning algorithms. This study provides a pathway toward trustworthy and accountable use of large language models in medicine.

## Introduction

### Background

Since the advent of pretrained language models, such as GPT [1] and bidirectional encoder representations from transformers (BERT) [2], in 2018, transformer-based [3] language models have revolutionized and popularized natural language processing (NLP). More recently, large language models (LLMs) [4,5] have demonstrated superior performance on zero-shot and few-shot tasks. Among LLMs, ChatGPT is favored by users due to its accessibility as well as its ability to produce grammatically correct and human-level answers in different domains. Since the release of ChatGPT in November 2022 by OpenAI, it has quickly gained significant attention within a few months. It has been widely discussed in the NLP community and other fields since then.

To balance the cost and efficiency of data annotation and train an LLM that better aligns with user intent in a helpful and safe manner, researchers used reinforcement learning from human feedback (RLHF) [6] to develop ChatGPT. RLHF uses a ranking-based human preference data set to train a reward model with which ChatGPT can be fine-tuned by proximal policy optimization [7]. As a result, ChatGPT can understand the meaning and intent behind user queries, which empowers ChatGPT to respond to queries in the most relevant and useful way. In addition to aligning with user intent, another factor that makes ChatGPT popular is its ability to handle a variety of tasks in different domains. The massive training corpus from the internet endows ChatGPT with the ability to learn the nuances of human language patterns. ChatGPT seems to be able to successfully generate human-level text content in all domains [8-12].

However, ChatGPT is a double-edged sword [13]. Misusing ChatGPT to generate human-like content can easily mislead users, resulting in wrong and potentially detrimental decisions. For example, malicious actors can use ChatGPT to generate a large number of fake reviews that damage the reputation of high-quality restaurants while falsely boosting the reputation of low-quality competitors. This is an example that can potentially harm consumers [14].

When using ChatGPT, some potential risks need to be considered. First of all, it may limit human creativity. ChatGPT has the ability to debug code or write essays for college students. It is important to consider whether ChatGPT will generate unique creative work or simply copy content from their training set. New York City public schools have banned ChatGPT.

What is more, ChatGPT has the ability to produce a text of surprising quality, which can deceive readers, and the end result is a dangerous accumulation of misinformation [15]. StackOverflow, a popular platform for coders and programmers, banned the use of ChatGPT-generated content because the average rate of correct answers from ChatGPT is too low and could cause significant harm to the site and the users who rely on it for accurate answers.

### Development of Language Models

The transformer-based language models have demonstrated a strong language modeling ability. Generally speaking, transformer-based language models are divided into 3 categories: encoder-based models (eg, BERT [2], Roberta [16], and Albert [17]), decoder-based models (eg, GPT [1] and GPT2 [18]), and encoder-decoder–based models (eg, Transformers [3], BART [19], and T5 [20]). In order to combine biomedical knowledge with language models, many researchers have added biomedical corpus for training [21-25]. Alsentzer et al [26] fine-tuned the publicly released BERT model on the Medical Information Mart for Intensive Care (MIMIC) data set [27] and demonstrated good performance on natural language inference and named entity recognition tasks. Lee et al [28] fine-tuned BERT on the PubMed data set, and it performed well on biomedical named entity recognition, biomedical relation extraction, and biomedical question-answering tasks. Based on the backbone of GPT2 [18], Luo et al [29] continued pretraining on the biomedical data set and showed superior performance on 6 biomedical NLP tasks. Other innovative applications include ClinicalRadioBERT [30] and SciEdBERT [31].

In recent years, decoder-based LLMs have demonstrated excellent performance on a variety of tasks [9,11,32,33]. Compared with previous language models, LLMs contain a large number of trainable parameters; for example, GPT-3 contains 175 billion parameters. The increased model size of GPT-3 makes it more powerful than previous models, boosting its language ability to near human levels in medical applications [34]. ChatGPT belongs to the GPT-3.5 series, which is fine-tuned based on RLHF. Previous research has shown that ChatGPT can achieve a passing score equivalent to that of a third-year medical student on a medical question-answering task [35].

ChatGPT has also demonstrated a strong understanding of high-stakes medical domains, including specialties such as radiation oncology [33]. Medical information typically requires rigorous validation. Indeed, false medical-related information generated by ChatGPT can easily lead to misjudgment of the developmental trend of diseases, delay the treatment process, or negatively affect the life and health of patients [36].

However, ChatGPT lacks the knowledge and expertise necessary to accurately and adequately convey complex scientific concepts and information. For example, human medical writers cannot yet be fully replaced because ChatGPT does not have the same level of understanding and expertise in the medical field [37]. To prevent the misuse use of ChatGPT to generate medical texts and avoid the potential risks of using ChatGPT, this study focuses on the detection of ChatGPT-generated text for the medical domain. We collected both publicly available expert-generated medical content and ChatGPT-generated content through the OpenAI interface. This study seeks to answer 2 questions: (1) What is the difference between medical content written by humans and that generated by ChatGPT? (2) Can we use machine learning methods to detect whether medical content is written by human experts or ChatGPT?

In this work, we make the following contributions to academia and industry:

- We construct 2 data sets to analyze the difference between ChatGPT-generated and human-generated medical text. We have published these 2 data sets to facilitate further analysis and research on ChatGPT for researchers.
- In this paper, we conducted a language analysis of medical content written by humans and that generated by ChatGPT. From the analysis results, we can grasp the difference between ChatGPT and humans in constructing medical content.
- We built a variety of machine learning models to detect text samples generated by humans and ChatGPT and explained and visualized the model structures.

In summary, this study is among the first efforts to qualitatively and quantitatively analyze and categorize differences between medical text generated by human experts and artificial intelligence–generated content (AIGC). We believe this work can spur further research in this direction and provide pathways toward responsible AIGC in medicine.

## Methods

### Data Set Construction

To analyze and discriminate human- and ChatGPT-generated medical texts, we constructed the following 2 data sets:

- Medical abstract data set: This original data set came from the work of Schopf et al [38] and involves digestive system diseases, cardiovascular diseases, neoplasms, nervous system diseases, and general pathological conditions.
- Radiology report data set: This original data set came from the work of Johnson et al [27], and only a subset of radiology reports were selected to build our radiology report data set.

**Figure 1:**
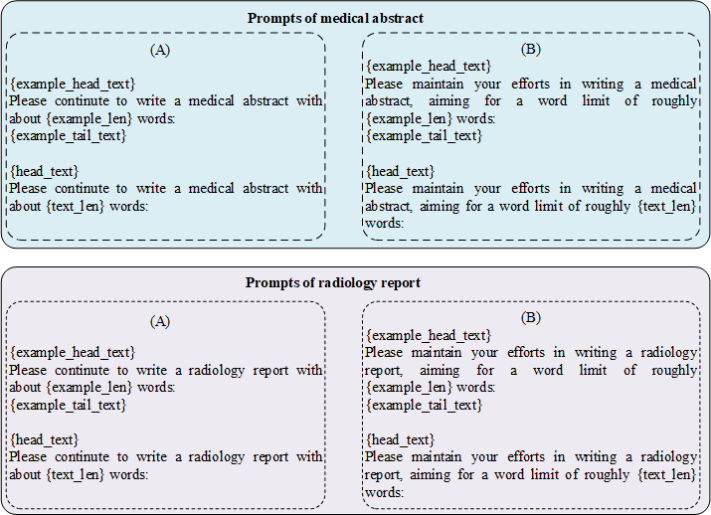
Prompts for building the ChatGPT-generated medical abstract and radiology report data sets.

Both the medical abstract and radiology report data sets are in English. We sampled 2200 text samples from the medical abstract and radiology report data sets as medical texts written by humans. In order to guide ChatGPT to generate medical content, we adopted the method of text continuation with demonstration instead of rephrasing [14] or query [39] with in-context learning because text continuation can produce more human-like text. The prompts used to generate medical abstract and radiology report data sets are shown in Figure 1. We used 2 different prompts to generate ChatGPT texts. In order to avoid the influence of ChatGPT randomness, we generated 2 groups of texts for each prompt. We randomly selected a sample (excluding the sample itself) from the data set as a demonstration. Finally, we obtained medical abstract and radiology report data sets containing 11,000 samples. According to the 2 different prompts and 2 different random groupings, these 11,000 samples can form 4 groups of data, each containing the same 2200 samples written by humans and 8800 samples generated by ChatGPT with one of the prompts and one of the random groups.

### Linguistic Analysis

We performed linguistic analysis of the medical content generated by humans and ChatGPT, including vocabulary and sentence feature analysis, part-of-speech (POS) analysis, dependency parsing, sentiment analysis, and text perplexity.

Vocabulary and sentence feature analysis illuminates the differences in the statistical characteristics of the words and sentences constructed by humans and ChatGPT when generating medical texts. We used the Natural Language Toolkit [40] to perform POS analysis. Dependency parsing is a technique that analyzes the grammatical structure of a sentence by identifying the dependencies between the words of the sentence. We applied CoreNLP (Stanford NLP Group) [41] for dependency parsing and compared the proportions of different dependency relationships and their corresponding dependency distances. We applied a pretrained sentiment analysis model [42] to conduct sentiment analysis for both the medical abstract and radiology report data sets. Perplexity is often used as a metric to evaluate the performance of a language model, with lower perplexity indicating that the language model is more confident in its predictions. We used the BioGPT [29] model to compute the perplexity of the human-written and ChatGPT-generated medical text.

### Detecting ChatGPT-Generated Text

Text content generated by the LLM has become popular on the internet. Since most of the content generated by LLMs is text with a fixed language pattern and language style, when a large number of generated text content appears, it will not be conducive to human active creation and can cause panic if incorrect medical text is generated. We used a variety of methods to detect medical texts generated by ChatGPT to reduce the potential risks to society caused by improper or malicious use of language models.

First, we divided the medical abstract and radiology report data sets into a training set, test set, and validation set at a ratio of 7:2:1, respectively. Then, we used a variety of algorithms to train the model with the training set, selected the best model parameters through the validation set, and finally calculated the metrics using the test set. The following models were used:

- Perplexity-classification (Perplexity-CLS): As text written by humans usually has higher text perplexity than that generated by ChatGPT, an intuitive idea was to find an optimal perplexity threshold to detect medical text generated by ChatGPT. This idea is the same as GPTZero [43], but our data is medical-related text, so we used BioGPT [29] as a language model to calculate text perplexity. We found the optimal perplexity threshold of the validation set and calculated the metrics on the test set.
- Classification and Regression Trees (CART): CART is a classic decision tree algorithm that tree uses the Gini index as the measure of feature division. We vectorized the samples through term frequency–inverse document frequency, and for convenience of visualization, we set the maximum depth of the tree to 4.
- XGBoost [44]: XGBoost is an ensemble learning method, and we set the maximum depth for base learners as 4 and vectorize the samples by term frequency–inverse document frequency.
- BERT [2]: BERT is a pretrained language model. We fine-tuned our medical text based on bert-base-cased [45].

In addition, we analyzed the CART, XGBoost, and BERT models to explore which features of the text help to detect text generated by ChatGPT.

### Ethical Considerations and Data Usage

In this study, we evaluated the proposed method on two medical datasets: medical abstracts describing patients’ conditions and radiology reports from the MIMIC-III dataset. Both datasets are extracted from publicly available sources. According to Luo et al [29], the free texts (including radiology reports) in the MIMIC-III dataset have been deidentified in accordance with Health Insurance Portability and Accountability Act (HIPAA) standards, using an existing, rigorously evaluated system [46]. Using publicly available and fully deidentified data for research purposes aligns with the waiver of human subjects protection issued by the Department of Health and Human Services (45 CFR 46.104) [47], which states that studies utilizing publicly available, anonymized data may not require formal ethics approval. The Institutional Review Board of Mass General Brigham negates the necessity for review for research exempted under 45 CFR 46.104 [48]. The datasets collected were strictly used for research purposes limited within this work, focusing on method development and validation without compromising individual privacy. In conclusion, this research adheres to the ethical guidelines and policies set forth by the Institutional Review Board of Mass General Brigham, ensuring that all data usage is responsible, respectful of privacy, and within the bounds of academic research.

## Results

### Linguistic Analysis

We conducted linguistic analysis of 2200 human-written samples and 8800 ChatGPT-generated samples from the medical abstract and radiology report data sets.

#### Vocabulary and Sentence Analysis

As shown in Table 1, from the perspective of statistical characteristics, the main differences between human-written medical text and medical text generated by ChatGPT involved the vocabulary and stem. Human-written medical text vocabulary size and the number of stems were significantly larger than those of ChatGPT-generated medical text. This suggests that the content and expression of medical texts written by humans are more diverse, which is more in line with the actual patient situation, while texts generated by ChatGPT are more inclined to use commonly used words to express common situations.

**Table 1:** Vocabulary and sentence analysis of human- and ChatGPT-generated text in the medical abstract and radiology report data sets.

		Vocabulary^a^	Word stems^b^	Sentences per sample, mean (SD)	Sentence length (words), mean (SD)	Text length (words), mean (SD)
**Medical abstract data set**						
	Human	22,889	16,195	8.7 (2.3)	16.2 (10.5)	146.3 (19.4)
	ChatGPT	15,782	11,120	10.4 (2.5)	15.7 (8.3)	168.6 (27.2)
**Radiology report data set**						
	Human	11,095	8396	12.7 (2.6)	10.4 (6.9)	135.9 (19.5)
	ChatGPT	7733	5774	12.5 (3.2)	10.2 (5.7)	130.5 (31.3)

^a^Total number of unique words across all samples.

^b^Total number of unique word stems across all samples.

#### Part-of-Speech Analysis

The results of POS analysis are shown in Table 2. ChatGPT used more words from the following categories: noun, singular or mass; determiner; noun, plural; and coordinating conjunction. ChatGPT used fewer cardinal digits and adverbs.

Frequent use of nouns (singular or mass and plural) tends to indicate that the text is more argumentative, showing information and objectivity [49]. The high proportion of coordinating conjunctions and determiners in ChatGPT-generated text indicated that the structure of the medical text and the relationship between causality, progression, or contrast was clear. At the same time, a large number of cardinal digits and adverbs appeared in medical texts written by humans, indicating that the expressions were more specific rather than general. For example, doctors will use specific numbers to describe the size of tumors.

**Table 2:** Top 20 parts-of-speech comparison between human-written and ChatGPT-generated text in the medical abstract and radiology report data sets.

Category	Medical abstract data set			Radiology report data set		
	Human (n=294,700), n (%)	ChatGPT (n=1,358,297), n (%)	Human (n=263,097), n (%)		ChatGPT (n=1,047,319), n (%)	
Noun, singular or mass	66,052 (22.4)	315,326 (23.2)	65,678 (25)		265,415 (25.3)	
Adjective	45,157 (15.3)	209,179 (15.4)	48,690 (18.5)		196,195 (18.7)	
Preposition or subordinating conjunction	42,496 (14.4)	182,029 (13.4)	25,070 (9.5)		96,548 (9.2)	
Determiner	25,947 (8.8)	127,371 (9.4)	22,720 (8.6)		106,668 (10.2)	
Noun, plural	23,918 (8.1)	122,615 (9)	9511 (3.6)		57,902 (5.5)	
Coordinating conjunction	11,292 (3.8)	56,301 (4.1)	7305 (2.8)		41,160 (3.9)	
Cardinal digit	10,718 (3.6)	25,053 (1.8)	4132 (1.6)		8881 (0.8)	
Verb, past tense	10,613 (3.6)	47,084 (3.5)	3000 (1.1)		8839 (0.8)	
Verb, past participle	10,517 (3.6)	44,381 (3.3)	8935 (3.4)		40,067 (3.8)	
Proper noun, singular	10,075 (3.4)	51,644 (3.8)	30,463 (11.6)		90,531 (8.6)	
Adverb	7311 (2.5)	22,606 (1.7)	6142 (2.3)		14,082 (1.3)	
To	4646 (1.6)	26,474 (1.9)	2424 (0.9)		10,533 (1)	
Verb, base form	4569 (1.6)	27,916 (2.1)	2527 (1)		8501 (0.8)	
Verb, third person singular present	3928 (1.3)	20,371 (1.5)	10,877 (4.1)		40,737 (3.9)	
Verb, gerund or present participle	3760 (1.3)	30,265 (2.2)	2492 (0.9)		9304 (0.9)	
Verb, nonthird person singular present	3237 (1.1)	13,166 (1)	3950 (1.5)		25,160 (2.4)	
Personal pronoun; possessive pronoun	1681 (0.6)	5775 (0.4)	—^a^		—	
Modal	1663 (0.6)	6717 (0.5)	970 (0.4)		2023 (0.2)	
Adjective, comparative	1311 (0.4)	4724 (0.3)	1401 (0.5)		3114 (0.3)	
Wh-determiner	937 (0.3)	2793 (0.2)	655 (0.2)		1257 (0.1)	
Existential there	—	—	3925 (1.5)		11075 (1.1)	

^a^Not in the top 20 parts-of-speech.

#### Dependency Parsing

The results of dependency parsing are shown in Table 3 and Table 4. As shown in Table 3, the comparison of dependencies exhibited similar characteristics to the POS analysis, where ChatGPT used more determiner, conjunct, coordination, and direct object relations while using fewer numeric modifiers and adverbial modifiers. For dependency distance, ChatGPT had obviously shorter conjuncts, coordinations, and nominal subjects, which made the text generated by ChatGPT more logical and fluent.

**Table 3:** Top 20 dependencies comparison between human-written and ChatGPT-generated text in the medical abstract and radiology report data sets.

Category	Medical abstract data set		Radiology report data set	
	Human (n=329,173), n (%)	ChatGPT (n=1,515,865), n (%)	Human (n=298,214), n (%)	ChatGPT (n=1,191,518), n (%)
Adjectival modifier	42,577 (12.9)	200,664 (13.2)	45,094 (15.1)	180,051 (15.1)
Case marking	42,056 (12.8)	183,711 (12.1)	25,813 (8.7)	104,999 (8.8)
Nominal modifier	40,288 (12.2)	176,319 (11.6)	24,137 (8.1)	95,435 (8)
Punctuation	35,433 (10.8)	157,984 (10.4)	46,980 (15.8)	179,102 (15)
Determiner	24,319 (7.4)	123,870 (8.2)	18,988 (6.4)	78,792 (6.6)
Compound	19,196 (5.8)	94,106 (6.2)	17,106 (5.7)	66,782 (5.6)
Root of the sentence	15,502 (4.7)	77,530 (5.1)	24,871 (8.3)	99,851 (8.4)
Conjunct	13,844 (4.2)	66,165 (4.4)	8811 (3)	46,438 (3.9)
Nominal subject	12,623 (3.8)	59,305 (3.9)	11,598 (3.9)	46,113 (3.9)
Coordination	11,633 (3.5)	56,862 (3.8)	7740 (2.6)	41,696 (3.5)
Direct object	9069 (2.8)	65,687 (4.3)	3788 (1.3)	16,762 (1.4)
Numeric modifier	8380 (2.5)	22,424 (1.5)	3013 (1)	8484 (0.7)
Adverbial modifier	7548 (2.3)	25,025 (1.7)	6646 (2.2)	15,820 (1.3)
Passive auxiliary	5942 (1.8)	23,818 (1.6)	4981 (1.7)	26,559 (2.2)
Marker	4723 (1.4)	31,131 (2.1)	—^a^	—
Dependent	4357 (1.3)	10,253 (0.7)	16,440 (5.5)	49,178 (4.1)
Copula	4082 (1.2)	15,479 (1)	5236 (1.8)	18,305 (1.5)
Clausal modifier of a noun	3451 (1)	23,387 (1.5)	2504 (0.8)	10,485 (0.9)
Auxiliary	3149 (1)	10,584 (0.7)	—	—
Passive nominal subject	5522 (1.7)	22,650 (1.5)	4717 (1.6)	26,035 (2.2)
Negation modifier	—	—	4156 (1.4)	29,109 (2.4)
Expletive	—	—	3927 (1.3)	11,069 (0.9)

^a^Not in the top 20 dependencies.

**Table 4:** Top 20 dependency distances comparison between human-written and ChatGPT-generated text in the medical abstract and radiology report data sets.

Category	Medical abstract data set		Radiology report data set	
	Human (words)	ChatGPT (words)	Human (words)	ChatGPT (words)
Adjectival modifier	1.5	1.4	1.7	1.6
Case marking	2.2	2.2	2.5	2.4
Nominal modifier	4.2	4.1	4.2	4.0
Punctuation	8.5	8.7	5.6	5.5
Determiner	1.8	1.7	2.1	2.0
Compound	1.3	1.2	1.5	1.4
Root of the sentence	7.3	5.9	3.6	4.0
Conjunct	5.9	4.7	4.5	3.6
Nominal subject	3.9	3.0	3.2	2.8
Coordination	3.7	2.9	2.4	1.8
Direct object	2.5	2.4	2.5	2.6
Numeric modifier	1.3	1.2	1.4	1.3
Adverbial modifier	2.2	2.8	1.7	2.1
Passive auxiliary	1.2	1.1	1.2	1.1
Marker	3.5	2.4	—^a^	—
Dependent	4.8	4.7	3.7	3.6
Copula	2.0	2.4	1.7	1.6
Clausal modifier of noun	2.3	2.5	2.3	2.4
Auxiliary	1.9	1.7	—	—
Passive nominal subject	6.1	5.2	3.8	3.8
Negation modifier	—	—	1.7	1.8
Expletive	—	—	1.3	1.1

^a^Not in the top 20 dependency distances.

#### Sentiment Analysis

The results of sentiment analysis are shown in Table 5. Most of the medical texts written by humans or those generated by ChatGPT had neutral sentiments. It should be noted that the proportion of negative sentiments in text written by humans was significantly higher than that in text generated by ChatGPT, while the proportion of positive sentiments in text written by humans was significantly lower than that in text generated by ChatGPT. This may be because ChatGPT has added a special mechanism to carefully filter the original training data set to ensure any violent or sexual content is removed, making the generated text more neutral or positive.

**Table 5:** Sentiment comparison between human-written and ChatGPT-generated text in the medical abstract and radiology report data sets

Sentiment	Medical abstract data set			Radiology report data set	
	Human (n=2200), n (%)	ChatGPT (n=8800), n (%)	Human (n=2200), n (%)		ChatGPT (n=8800), n (%)
Negative	432 (19.6)	1205 (13.7)	204 (9.3)		493 (5.6)
Neutral	1588 (72.2)	5822 (66.2)	1942 (88.3)		7738 (87.9)
Positive	180 (8.2)	1773 (20.2)	54 (2.5)		569 (6.5)

#### Text Perplexity

**Figure 2:**
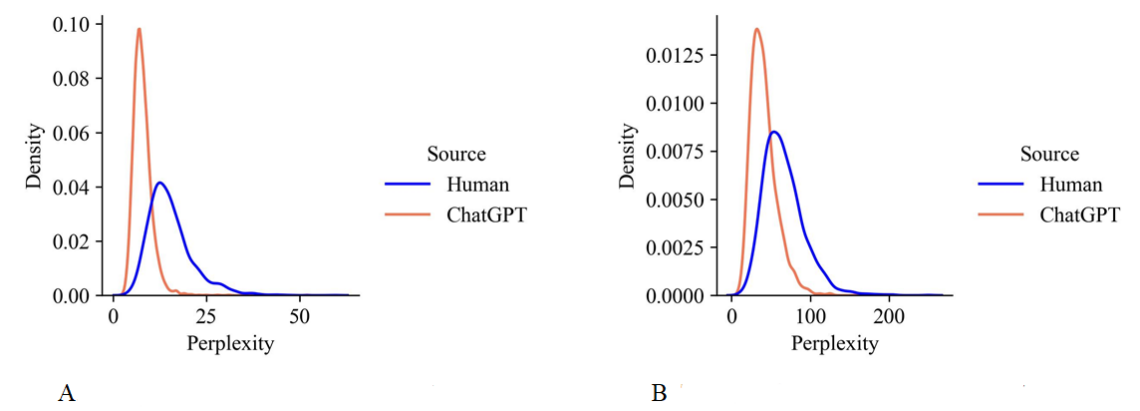
Text perplexity of human-written and ChatGPT-generated (A) medical abstracts and (B) radiology reports.

The results of text perplexity are shown in Figure 2. It can be observed that for both medical abstract and radiation report data sets, the perplexity of text generated by ChatGPT was significantly lower than that of text written by humans. ChatGPT captures common patterns and structures in the training corpus and is very good at replicating them. Therefore, the text generated by ChatGPT has relatively low perplexity. Humans can express themselves in a variety of ways, depending on the intellectual context, the condition of the patient, and other factors, which may make BioGPT more difficult to predict. Therefore, human-written text had a higher perplexity and wider distribution.

Through the above analysis, we identified the main differences between the human-written and ChatGPT-generated medical text as the following: (1) medical texts written by humans were more diverse, while medical texts generated by ChatGPT were more common; (2) medical texts generated by ChatGPT had better logic and fluency; (3) medical texts written by humans contained more specific values, and text content was more specific; (4) medical texts generated by ChatGPT were more neutral and positive; and (5) ChatGPT had lower text perplexity because it is good at replicating common expression patterns and sentence structures.

### Detecting ChatGPT-Generated Text

The results of detecting ChatGPT-generated medical text are shown in Table 6. The results shown in Table 6 are the average of the accuracy across the 4 groups. Compared with similar works [14,39] for detecting ChatGPT-generated content, our detection performance showed much higher accuracy. Since Perplexity-CLS is an unsupervised learning method, it was less effective than other methods. XGBoost integrates the results of multiple decision trees, so it worked better than CART with a single decision tree. The pretrained BERT model easily recognized differences in the logical structure and language style of medical texts written by humans and those generated by ChatGPT, thus achieving the best performance.

**Table 6:** Results of detecting ChatGPT-generated medical text in the medical abstract and radiology data sets.

		Accuracy		Precision		Recall	*F*_1_ score
**Perplexity-CLS^a^, mean (SD)**							
	Medical abstract		0.847 (0.014)		0.849 (0.015)	0.847 (0.014)	0.847 (0.014)
	Radiology report		0.743 (0.011)		0.756 (0.015)	0.743 (0.011)	0.74 (0.011)
**CART^b^, mean (SD)**							
	Medical abstract		0.869 (0.019)		0.888 (0.012)	0.867 (0.019)	0.867 (0.02)
	Radiology report		0.831 (0.004)		0.837 (0.007)	0.831 (0.004)	0.83 (0.005)
**XGBoost, mean (SD)**							
	Medical abstract		0.957 (0.007)		0.958 (0.006)	0.957 (0.007)	0.957 (0.007)
	Radiology report		0.924 (0.007)		0.925 (0.006)	0.924 (0.007)	0.924 (0.007)
**BERT^c^, mean (SD)**							
	Medical abstract		0.982 (0.003)		0.982 (0.003)	0.982 (0.003)	0.982 (0.003)
	Radiology report		0.956 (0.033)		0.957 (0.032)	0.956 (0.033)	0.956 (0.033)

^a^Perplexity-CLS: Perplexity-classification.

^b^CART: classification and regression trees.

^c^BERT: bidirectional encoder representations from transformers.

**Figure 3:**
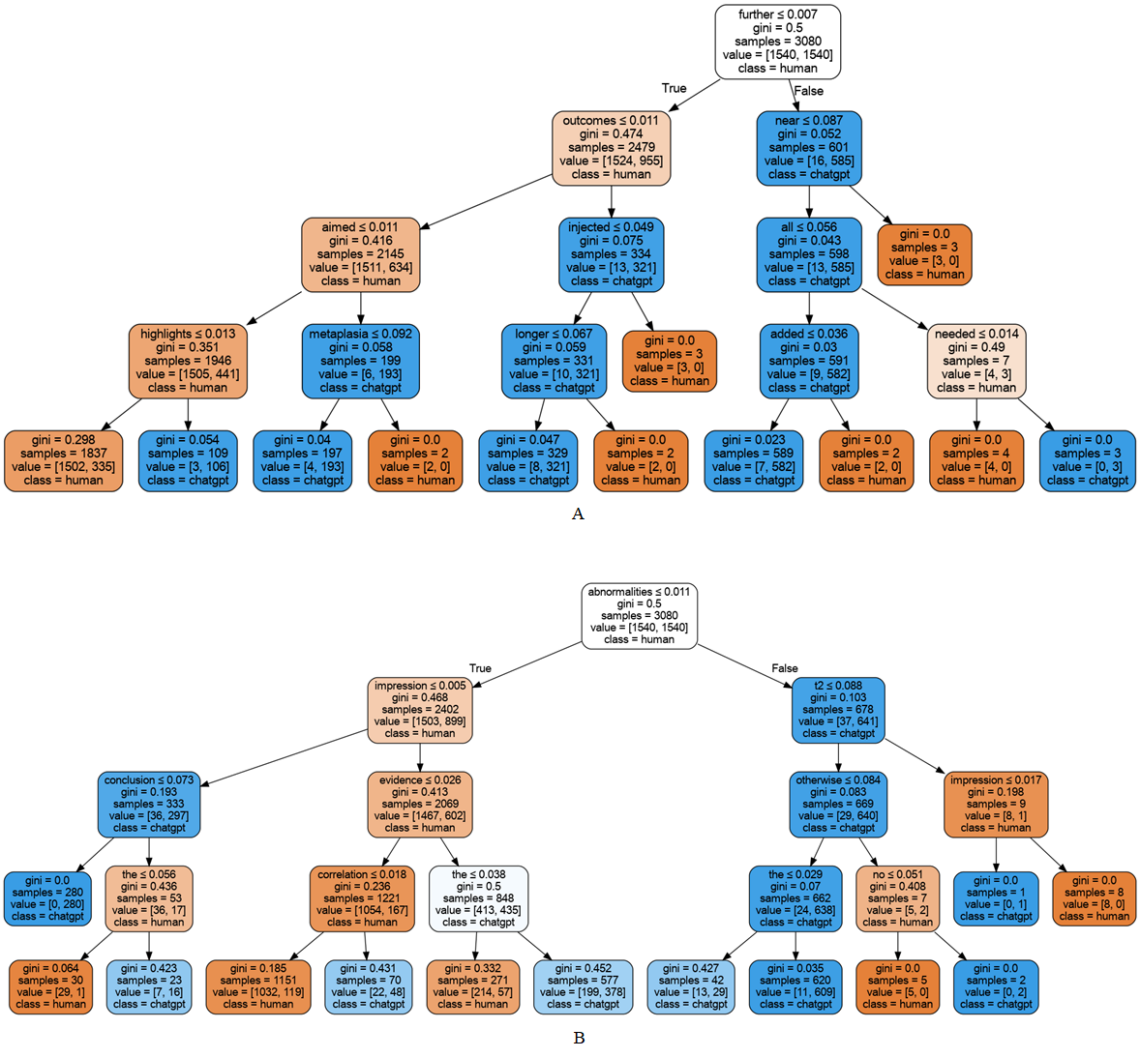
Visualization of the CART model for the (A) medical abstracts and (B) radiology reports data sets. CART: classification and regression trees.

Figure 3 presents the visualization of the CART model of the 2 data sets. Through the decision tree with depth 4, the text generated by ChatGPT was detected well. We calculated the contribution of each feature of the XGBoost model, and the top 15 most important features are shown in Tables 7 and 8. Comparing Figure 3 and Table 7, we can see that the decision tree nodes are similar. For example, in the medical abstract data set, “further,” “outcomes,” “highlights,” and “aimed” are important features of the CART and XGBoost models.

**Figure 4:**
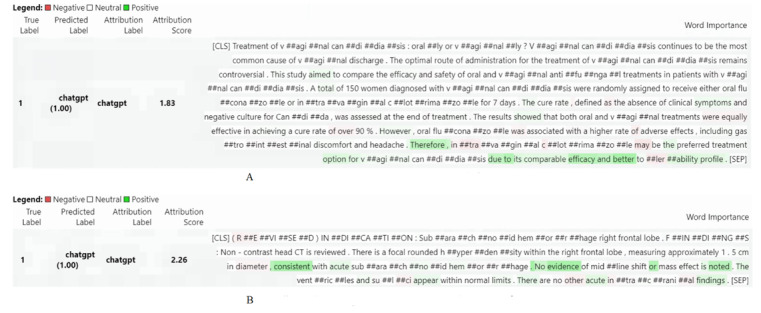
Visualization of the features of the samples for the (A) medical abstracts and (B) radiology reports data sets using BERT. BERT: bidirectional encoder representations from transformers.

In addition to visualizing the global features of CART and XGBoost, we also used the transformers-interpret toolkit [50] to visualize the local features of the samples, and the results are shown in Figure 4. For BERT, conjuncts were important features for detecting ChatGPT-generated text (eg, “due to,” “therefore,” and “or”). In addition, the important features of BERT were similar to those of XGboost. For example, “evidence,” “findings,” and “acute” were important features in the radiology report data set for detecting medical text generated by ChatGPT.

**Table 7:** Important features of the medical abstract data set.

Feature	Importance (*F* score)
Outcomes	24
Further	24
Findings	21
Potential	19
This	16
The	15
Highlights	15
Management	14
Aimed	14
Study	12
May	12
Report	10
Rare	10
Crucial	10
Results	9

**Table 8:** Important features of the radiology reports data set.

Feature	Importance (*F* score)
The	74
Impression	48
There	31
No	25
Acute	25
Evidence	21
Findings	20
Significant	16
Correlation	15
Conclusion	15
Identified	14
Left	13
Previous	12
Consistent	11
Observed	10

## Discussion

### Principal Results

In this paper, we focused on analyzing the differences between medical texts written by humans and those generated by ChatGPT and designed machine learning algorithms to detect medical texts generated by ChatGPT. The results showed that medical texts generated by ChatGPT were more fluent and logical but had low information content. In contrast, medical texts written by humans were more diverse and specific. Such differences led to the potential discriminability between these two.

ChatGPT simply imitates human language and uses general information content, which makes it challenging to generate text on personalized treatment and conditions with high intersubject heterogeneity. Such an issue may potentially lead to decreased patient care quality throughout the whole clinical workflow. For the purpose of medical education, AIGC has led to much awareness and concerns over its possible misuse. Students and trainees could use ChatGPT for assignments and exams. In addition, using such tools can hinder the students’ learning process, especially at the current stage, where curriculum design has not been updated accordingly [51]. Finally, as more patients rely on internet searches to seek medical advice, it is important to mark the AIGC, especially that related to medicine, with “Generated by AIGC” labels. By doing so, we can further deal with potential issues in ChatGPT-generated text caused by system-wide errors and algorithm biases, such as the “hallucination effect” of generative modeling and outdated information sources.

In order to mitigate and control the potential harm caused by medical AIGC, we developed algorithms to identify content generated by ChatGPT. Although ChatGPT can generate human-like text, due to the differences in language style and content, the text written by ChatGPT can still be accurately detected by designing machine learning algorithms, and the *F*_1_ score exceeded 95%. This study provides a pathway toward trustworthy and accountable use of LLMs in medicine.

### Limitations

This paper is dedicated to analyzing the differences between medical texts written by humans and those generated by ChatGPT. We developed various machine-learning algorithms to distinguish the two. However, our work has some limitations. First, this paper only analyzes medical abstracts and radiology reports; however, there exist various other types of medical texts, and these 2 types of medical texts are just examples. Second, ChatGPT is a model that can handle multiple languages, but the data sets we used were only in English. Additionally, we only used ChatGPT as an example to analyze the difference between medical texts generated by an LLM and medical texts written by humans; however, more advanced LLMs, such as GPT-4 and other open-source models, have emerged. It will be part of our future work to analyze more language styles generated by other LLMs and summarize their general language construction rules.

### Conclusions

In general, for artificial intelligence (AI) to realize its full potential in medicine, we should not rush into its implementation but advocate for its careful introduction and open debate about its risks and benefits. First, human medical writers will be responsible for ensuring the accuracy and completeness of the information communicated and for complying with ethical and regulatory guidelines. However, ChatGPT cannot be held responsible. Second, training an LLM requires a huge amount of data, but the quality of the data is difficult to guarantee, so the trained ChatGPT is biased. For example, ChatGPT can provide biased output and perpetuate sexist stereotypes [52]. Third, use of ChatGPT may lead to private information leakage. This may be because the LLM remembers personal privacy information in the training set [53]. What is more, the legal framework must be considered. Who shall be held accountable when an AI doctor makes an inevitable mistake? ChatGPT cannot be held accountable for its work, and there is no legal framework to determine who owns the rights to AI-generated work [15].

The medical field is a field related to human health and life. We provided a simple demonstration to identify ChatGPT-generated medical content, which can help reduce the harm caused to humans by erroneous and incomplete ChatGPT-generated information. Assessing and mitigating the risks associated with LLMs and their potential harm is a complex and interdisciplinary challenge that requires combining knowledge from various fields to drive the healthy development of LLMs.

## Abbreviations

AI: artificial intelligence
AIGC: artificial intelligence–generated content
BERT: bidirectional encoder representations from transformers
CART: classification and regression trees
HIPAA: Health Insurance Portability and Accountability Act
LLM: large language model
MIMIC: Medical Information Mart for Intensive Care
NLP: natural language processing
Perplexity-CLS: Perplexity-classification
POS: part-of-speech
RLHF: reinforcement learning from human feedback

## Data Availability

The data and code generated in this study are available on GitHub [54].

## References

[1] Radford A, Narasimhan K, Salimans T, Sutskever I (2018). Improving language understanding by generative pre-training. OpenAI.

[2] Devlin J, Chang MW, Lee K, Toutanova K (2019). Bert: pre-training of deep bidirectional transformers for language understanding.

[3] Vaswani A, Shazeer N, Parmar N, Uszkoreit J, Jones L, Gomez AN, Kaiser L, Polosukhin I (2017). Attention is all you need. Adv Neural Inf Process Syst.

[4] Brown T, Mann B, Ryder N, Subbiah M, Kaplan JD, Dhariwal P, Neelakantan A, Shyam P, Sastry G, Askell A (2020). Language models are few-shot learners. Adv Neural Inf Process Syst.

[5] Ouyang L, Wu J, Jiang X, Almeida D, Wainwright CL, Mishkin P, Zhang C, Agarwal S, Slama K, Ray A Training language models to follow instructions with human feedback. arXiv.

[6] Christiano PF, Leike J, Brown T, Martic M, Legg S, Amodei D (2017). Deep reinforcement learning from human preferences. Adv Neural Inf Process Syst.

[7] Schulman J, Wolski F, Dhariwal P, Radford A, Klimov O Proximal policy optimization algorithms. arXiv.

[8] Guan Z, Wu Z, Liu Z, Wu D, Ren H, Li Q, Li X, Liu N Cohortgpt: an enhanced gpt for participant recruitment in clinical study. arXiv.

[9] Dai H, Liu Z, Liao W, Huang X, Wu Z, Zhao L, Liu W, Liu N, Li S, Zhu D AugGPT: leveraging ChatGPT for text data augmentation. arXiv.

[10] Ma C, Wu Z, Wang J, Xu S, Wei Y, Liu Z, Jiang X, Guo L, Cai X, Zhang S, Zhang T, Zhu D, Shen D, Liu T, Li X ImpressionGPT: an iterative optimizing framework for radiology report summarization with ChatGPT. arXiv.

[11] Liu Z, Yu X, Zhang L, Wu Z, Cao C, Dai H, Zhao L, Liu W, Shen D, Li Q Deid-GPT: zero-shot medical text de-identification by GPT-4. arXiv.

[12] Shi Y, Xu S, Liu Z, Liu T, Li X, Liu N MedEdit: model editing for medical question answering with external knowledge bases. arXiv.

[13] Hisan UK, Amri MM (2023). ChatGPT and medical education: a double-edged sword. J Educ Pedagog.

[14] Mitrović S, Andreoletti D, Ayoub O ChatGPT or human? Detect and explain. Explaining decisions of machine learning model for detecting short ChatGPT-generated text. arXiv.

[15] Homolak J (2023). Opportunities and risks of ChatGPT in medicine, science, and academic publishing: a modern Promethean dilemma. Croat Med J.

[16] Liu Y, Ott M, Goyal N, Du J, Joshi M, Chen D, Levy O, Lewis M, Zettlemoyer L, Stoyanov V RoBERTa: a robustly pptimized BERT pretraining approach. arXiv.

[17] Lan Z, Chen M, Goodman S, Gimpel K, Sharma P, Soricut R ALBERT: A lite BERT for self-supervised learning of language representations. arXiv.

[18] Radford A, Wu J, Child R, Luan D, Amodei D, Sutskever I (2019). Language models are unsupervised multitask learners. Semantic Scholar.

[19] Lewis M, Liu Y, Goyal N, Ghazvininejad M, Mohamed A, Levy O, Stoyanov V, Zettlemoyer L (2020). BART: denoising sequence-to-sequence pre-training for natural language generation, translation, and comprehension.

[20] Raffel C, Shazeer N, Roberts A, Lee K, Narang S, Matena M, Zhou Y, Li W, Liu PJ (2020). Exploring the limits of transfer learning with a unified text-to-text transformer. J Mach Learn Res.

[21] Liao W, Liu Z, Dai H, Wu Z, Zhang Y, Huang X, Chen Y, Jiang X, Zhu D, Liu T Mask-guided BERT for few shot text classification. arXiv.

[22] Cai H, Liao W, Liu Z, Huang X, Zhang Y, Ding S, Li S, Li Q, Liu T, Li X Coarse-to-fine knowledge graph domain adaptation based on distantly-supervised iterative training. arXiv.

[23] Liu Z, He M, Jiang Z, Wu Z, Dai H, Zhang L, Luo S, Han T, Li X, Jiang X, Zhu D, Cai X, Ge B, Liu W, Liu J, Shen D, Liu T (2022). Survey on natural language processing in medical image analysis. Zhong Nan Da Xue Xue Bao Yi Xue Ban.

[24] Liu Y, Han T, Ma S, Zhang J, Yang Y, Tian J, He H, Li A, He M, Liu Z, Wu Z, Zhao L, Zhu D, Li X, Qiang N, Shen D, Liu T, Ge B (2023). Summary of ChatGPT-related research and perspective towards the future of large language models. Meta Radiology.

[25] Zhao L, Zhang L, Wu Z, Chen Y, Dai H, Yu X, Liu Z, Zhang T, Hu X, Jiang X, Li X, Zhu D, Shen D, Liu T (2023). When brain-inspired AI meets AGI. Meta Radiology.

[26] Alsentzer E, Murphy JR, Boag W, Weng WH, Jin D, Naumann T, McDermott M Publicly available clinical BERT embeddings. arXiv.

[27] Johnson AE, Pollard TJ, Shen L, Lehman LH, Feng M, Ghassemi M, Moody B, Szolovits P, Celi LA, Mark RG (2016). MIMIC-III, a freely accessible critical care database. Sci Data.

[28] Lee J, Yoon W, Kim S, Kim D, Kim S, So CH, Kang J (2020). BioBERT: a pre-trained biomedical language representation model for biomedical text mining. Bioinformatics.

[29] Luo R, Sun L, Xia Y, Qin T, Zhang S, Poon H, Liu TY (2022). BioGPT: generative pre-trained transformer for biomedical text generation and mining. Brief Bioinform.

[30] Rezayi S, Dai H, Zhao L, Wu Z, Hebbar A, Burns AH, Lin Z, Zhu D, Li Q, Liu W, Lian C, Cao X, Rekik I, Xu X, Cui Z (2022). Clinicalradiobert: Knowledge-infused few shot learning for clinical notes named entity recognition. Machine Learning in Medical Imaging. MLMI 2022. Lecture Notes in Computer Science.

[31] Liu Z, He X, Liu L, Liu T, Zhai X (2023). Context matters: a strategy to pre-train language model for science education. SSRN Journal.

[32] Liu Z, Zhong A, Li Y, Yang L, Ju C, Wu Z, Ma C, Shu P, Chen C, Kim S, Dai H, Zhao L, Zhu D, Liu J, Liu W, Shen D, Li X, Li Q, Liu T Radiology-GPT: a large language model for radiology. arXiv.

[33] Holmes J, Liu Z, Zhang L, Ding Y, Sio TT, McGee LA, Ashman JB, Li X, Liu T, Shen J, Liu W (2023). Evaluating large language models on a highly-specialized topic, radiation oncology physics. Front Oncol.

[34] Liu Z, Li Y, Shu P, Zhong A, Yang L, Ju C, Wu Z, Ma C, Luo J, Chen C, Kim S, Hu J, Dai H, Zhao L, Zhu D, Liu J, Liu W, Shen D, Liu T, Li Q, Li X Radiology-Llama2: best-in-class large language model for radiology. arXiv.

[35] Gilson A, Safranek CW, Huang T, Socrates V, Chi L, Taylor RA, Chartash D (2023). How does ChatGPT perform on the United States Medical Licensing Examination? The implications of large language models for medical education and knowledge assessment. JMIR Med Educ.

[36] Bickmore TW, Trinh H, Olafsson S, O'Leary TK, Asadi R, Rickles NM, Cruz R (2018). Patient and consumer safety risks when using conversational assistants for medical information: an observational study of Siri, Alexa, and Google Assistant. J Med Internet Res.

[37] Biswas S (2023). ChatGPT and the future of medical writing. Radiology.

[38] Schopf T, Braun D, Matthes F Evaluating unsupervised text classification: zero-shot and similarity-based approaches. arXiv.

[39] Guo B, Zhang X, Wang Z, Jiang M, Nie J, Ding Y, Yue J, Wu Y How close is chatgpt to human experts? Comparison corpus, evaluation, and detection. arXiv.

[40] Bird S, Klein E, Loper E (2009). Natural Language Processing with Python - Analyzing Text with the Natural Language Toolkit.

[41] Manning CD, Surdeanu M, Bauer J, Finkel J, Bethard SJ, McClosky D (2014). The Stanford CoreNLP natural language processing toolkit. Proceedings of the 52nd Annual Meeting of the Association for Computational Linguistics: System Demonstrations.

[42] Twitter-roBERTa-base for sentiment analysis. Hugging Face.

[43] GPTZero.

[44] Chen T, Guestrin C (2016). Xgboost: a scalable tree boosting system. Proceedings of the 22nd ACM SIGKDD International Conference on Knowledge Discovery and Data Mining.

[45] BERT base model (cased). Hugging Face.

[46] Neamatullah Ishna, Douglass Margaret M, Lehman Li-wei H, Reisner Andrew, Villarroel Mauricio, Long William J, Szolovits Peter, Moody George B, Mark Roger G, Clifford Gari D (2008). Automated de-identification of free-text medical records. BMC Med Inform Decis Mak.

[47] Department of Health and Human Services Section 46.104 Exempt Research. Code of Federal Regulation Title 45.

[48] Mass General Brigham Human Research Protection Program. https://www.massgeneralbrigham.org/en/research-and-innovation/for-researchers-and-collaborators/collaborators-and-sponsors/human-research-protection-program.

[49] Nagy W, Townsend D (2012). Words as tools: learning academic vocabulary as language acquisition. Read Res Q.

[50] Transformers-interpret. GitHub.

[51] Liu Z, Zhang L, Wu Z, Yu X, Cao C, Dai H, Liu N, Liu J, Liu W, Li Q (2023). Surviving chatgpt in healthcare. Front Radiol.

[52] The Lancet Digital Health (2023). ChatGPT: friend or foe?. Lancet Digit Health.

[53] Carlini N, Tramer F, Wallace E, Jagielski M, Herbert-Voss A, Lee K, Roberts A, Brown TB, Song D, Erlingsson U (2021). Extracting training data from large language models. Proceedings of the 30th USENIX Security Symposium.

[54] detect_ChatGPT. GitHub.

